# Evolution of speech-specific cognitive adaptations

**DOI:** 10.3389/fpsyg.2015.01505

**Published:** 2015-09-29

**Authors:** Bart de Boer

**Affiliations:** Artificial Intelligence Laboratory, Vrije Universiteit Brussel, Brussels, Belgium

**Keywords:** evolution of speech, combinatorial structure, language evolution, biology-culture co-evolution, language-specific selection

## Abstract

This paper argues that an evolutionary perspective is natural when investigating cognitive adaptations related to language. This is because there appears to be correspondence between traits that linguists consider interesting and traits that have undergone selective pressure related to language. The paper briefly reviews theoretical results that shed light on what kind of adaptations we can expect to have evolved and then reviews concrete work related to the evolution of adaptations for combinatorial speech. It turns out that there is as yet no strong direct evidence for cognitive traits that have undergone selection related to speech, but there is indirect evidence that indicates selection. However, the traits that may have undergone selection are expected to be continuously variable ones, rather than the discrete ones that linguists have focused on traditionally.

## Introduction

What properties of the brain make it language-ready? Many properties of the brain are needed, including “obvious” ones such as a supply of oxygen and nutrients. However, when cognitive scientists and linguists consider this question, they focus on properties that are to at least some extent unique to language and/or unique to humans (Hockett, 1960; Hauser et al., 2002). This is implicitly an evolutionary point of view, because what is investigated is defined in relation to what is found in related species. Here it is argued that even though the language-ready brain can be studied fruitfully without reference to its evolutionary history or without reference to comparable abilities in other species, keeping in mind the evolutionary perspective is important. After all, the behaviors and brain regions that are involved must either be similar to those of other apes, and if they are more different than would be expected from random drift, there must be an evolutionary reason, either related to language or not.

An evolutionary perspective may also help to resolve the debate about whether behaviors or mechanisms related to language are “language-specific” or “domain-general.” The problem is that one researcher’s “language-specific” is another researchers’ “domain-general,” as it is essentially arbitrary where one draws the line. From the evolutionary perspective this is even clearer as any cognitive mechanism involved in language must be based on an earlier one that was not. However, the evolutionary perspective may provide a way out, as the question of whether a trait has undergone selective pressure related to language is in principle amenable to empirical investigation (even though this may be very hard). Hence the question of whether a trait is domain-general or language-specific can be operationalized by asking whether it has undergone selective pressure related to language. In this paper, certain aspects of the language-ready brain related to speech will be considered from an evolutionary perspective. Speech is here defined as the physical signal that is used to convey language, and although this paper will focus on signals in the acoustic modality, most of what is said is true for sign language as well. Researchers with a naïve view of biology sometimes consider speech as a somewhat uninteresting process of externalization unrelated to the core properties of language (e.g.,Bolhuis et al., 2014). However, from an evolutionary perspective it is one of the most interesting aspects of language. There are three reasons for this. Firstly, speech is the aspect of language that is closest to the physical world and therefore the most likely to leave traces in the fossil record (de Boer, 2012; reviewed in, e.g., Fitch, 2010, section 2). Secondly, and related to this, speech has close analogies in other animals’ behaviors. Thirdly, speech has very interesting cognitive properties (defined more precisely below) that have been proposed by some researchers as direct precursors to syntax (Carstairs-McCarthy, 1999; Studdert-Kennedy, 2005).

Two cognitive properties that allow speech but that are not found in closely related primates are precise voluntary control over the larynx and extensive vocal imitation (Ackermann et al., 2014). This paper will focus on a third aspect: *combinatorial speech*, the ability to use a small set of learned building blocks that can be recombined into an unlimited number of utterances using learned rules. This ability to deal with combinatorial structure is the basis of the phonology and phonotactics of modern human languages. Before looking at evidence for language-specific selective pressure in cognitive traits for dealing with combinatorial structure, a brief theoretical discussion is necessary about what kinds of traits can evolve, and what can therefore be expected.

## Constraints on Evolution

An important constraint on evolution is that it needs to work with what is already there: selection works on variations in the population, and this variation is caused by randomness in transmission. However, transmission in complex organisms must be relatively high-fidelity and variation must therefore be small. Evolution will consequently be gradual. However, this appears to pose no important constraints on language evolution. Precursors of many of the prerequisites for language have been inferred for the latest common ancestor with the other apes (Fitch, 2010, chapter 6). In addition processes of analogous evolution observed in other groups of species show that traits required for language that are missing in the latest common ancestor can evolve relatively quickly, for instance vocal mimicry^1^ or (song) structure (Honda and Okanoya, 1999).

A more subtle constraint arises because language itself evolves culturally while humans evolve biologically. It has been argued that because culture changes much more quickly than biology, language provides an insufficiently stable target, and therefore arbitrary adaptations to it cannot evolve (Chater et al., 2009). Mathematical analysis shows that only the smallest stable learning biases need to evolve (Kirby et al., 2007; Smith, 2011; Thompson et al., 2012) because once a learning bias is in place cultural evolution will tend to amplify the effect of the bias, therefore masking the distinction between strong and weak biases, and thus eliminating any selective advantage of a stronger bias. If only small learning biases can evolve, it may be that these are too small to detect experimentally.

Nevertheless larger adaptations to culturally changing language can evolve through co-evolution between language and cognition (e.g., Deacon, 1997). This can happen when cultural evolution pushes the language to become more challenging for the learners (through expanding vocabulary, or through expanding the sound system, for instance). Biological evolution can then make a small adaptation (in the sense mentioned above). This will allow for cultural evolution to make the language even more complex than before, and through continuous co-evolution a large adaptation to language can eventually evolve. Candidate for such adaptations can be the ability to produce and perceive a large range of signals (de Boer, 2015) or the ability to learn large lexicons (de Boer, 2014). Such traits are by necessity continuously variable, whereas in general traits that are considered by linguists are discrete in nature, e.g., the ability to use recursion (Bolhuis et al., 2014), or the universals considered by Evans and Levinson (2009).

## Experimental Investigation

What evidence exists for adaptations dealing with combinatorial structure? The fact that languages can be analyzed as having combinatorial structure does not necessarily mean that this structure is also represented in the brain (Zuidema and de Boer, 2009). However, evidence from for instance speech errors (Meyer, 1992), treatment of loanwords (e.g., Vendelin and Peperkamp, 2006) or poetry (Maddieson, 2008) indicate that speakers are aware of the building blocks, even if these building blocks do not necessarily correspond to phonemes. Moreover, evidence from acquisition indicates that infants learn the building blocks and the structure of their language from a very young age, both in production of intonation (Mampe et al., 2009) or phonemes (e.g., Kuhl and Meltzoff, 1996) and in perception of phonemes (Maye et al., 2002; Kuhl, 2004). This indicates that there must be cognitive mechanisms that help in learning building blocks of speech, whereas there is no evidence that these mechanisms are present in other apes. On the other hand, evidence from the emerging sign languages ABSL (Sandler et al., 2011) and CTSL (Caselli et al., 2014) indicate that combinatorial structure emerges gradually in new human languages, and that full languages can exist without much combinatorial structure.

One way to operationalize the search for traits that have undergone selection related to language is to look for brain regions that react preferentially to language. There is good evidence that there are regions specialized for processing speech and phonetic cues (e.g., Leaver and Rauschecker, 2010) and that there are even regions specialized for phonotactics (Raettig and Kotz, 2008; Rossi et al., 2011). However, there is also evidence that the precise processing of phonotactic structure is influenced by literacy (Castro-Caldas et al., 1998). Incidentally, Vendelin and Peperkamp (2006) also found that orthography influences how loanwords are treated. This raises the question of how much of the observed specialization and behavior is due to acquisition, and how much of it is indicative of evolutionary selection due to speech. DNA studies may provide insight, but although our knowledge is expanding rapidly (Dediu, 2015), we are still far from being able to relate genetic evidence with speech, the vocal tract or the brain.

Another way to operationalize the search for language-related selection is to search for behaviors that behave differently for linguistic than for non-linguistic signals. For this one needs to conduct experiments using artificial signals or to have participants devise their own signals. This allows for the possibility to include the degree of resemblance to language as a condition in the experiments and therefore to detect specialization for language. In the context of language evolution, the first such experiments were done by Galantucci (2005), but these were mostly meant to investigate emergence of signals and their structure. Since then many experiments have been done to investigate language evolution in a laboratory setting (for reviews: Galantucci, 2009; Scott-Phillips and Kirby, 2010; Kirby et al., 2014). However, few of these experiments look at speech and signals, and those that do mainly focus on cultural processes of emergence of structure (e.g., Garrod et al., 2010; Roberts et al., 2015).

Verhoef et al. (2014) however have compared two different accounts of the emergence of combinatorial structure, one based on the communication-relevant needs for distinct signals, the other on cognitive principles of processing efficiency, and found that the way human participants create structure can best be explained by the latter account. Nonetheless, this study could not determine whether these cognitive processes were language-specific or not.

A study by van der Ham and de Boer (2015a) has looked at behavior of human participants in a distributional learning task of language-like stimuli and has explicitly tested whether reproduction behavior was as predicted by a domain-general learning mechanism or by a learning mechanism specialized for language. It was found that in this case, behavior could be explained by the domain-general mechanism. Another way to detect cognitive mechanisms that have undergone selective pressure related to speech is to look for mechanisms that behave differently for speech-like stimuli than for less speech-like stimuli. An experiment along these lines has compared category learning and reproduction in the acoustic, visual and tactile modalities (van der Ham and de Boer, 2015b) and found that humans are somewhat better in the tactile and acoustic modalities, but that there is no indication of strong specialization. Results so far therefore do not show unambiguous evidence that point to selective pressure related to language.

## Discussion

Although so far no cognitive traits that have undergone selective pressure related to speech have been identified, and although identifying the selective pressures that have shaped any trait is very difficult, nevertheless the evolutionary perspective can help structure research into the cognition of speech and language. After all, the intuitive notion of what cognitive traits are linguistically interesting corresponds to what traits have evolved under selective pressure for language. In addition the evolutionary perspective may help determine what kind of traits can have evolved and those may be rather different than the kind of traits linguists have traditionally focused on—less discrete and formal, more continuous and related to the function of language. Finally, the interdisciplinary approach that the evolutionary perspective entails has led to a number of promising new tools to investigate cognitive adaptations related to language.

### Conflict of Interest Statement

The author declares that the research was conducted in the absence of any commercial or financial relationships that could be construed as a potential conflict of interest.

## Footnotes

1 For example, whereas the Black-browed Reed Warbler (*Acrocephalus bistrigiceps*) mimics 2–5 species, the closely related Marsh Warbler (*Acrocephalus palustris*) mimics more than 100 (Hamao and Eda-Fujiwara, 2004). Nevertheless their cytochrome b (mtDNA) distance is only 10–11% (Leisler et al., 1997) whereas that between humans and chimpanzees is 15–16% (Castresana, 2001).
